# Tetanus outbreak in a sheep flock due to ear tagging

**DOI:** 10.1002/vms3.139

**Published:** 2018-12-13

**Authors:** Samad Lotfollahzadeh, Masoumeh Heydari, Mohammad Reza Mohebbi, Maryam Hashemian

**Affiliations:** ^1^ Department of Internal Medicine Faculty of Veterinary Medicine University of Tehran Tehran Iran

**Keywords:** ear tagging, penicillin, sheep, tetanus

## Abstract

Tetanus is an acute, often fatal, infectious neuromuscular disease in all farmed mammals caused by *Clostridium tetani*. The disease is sporadic but outbreaks of tetanus have been described, as a result of wound contaminated with spores of *C. tetani*, which sporulates to the vegetative form and produce toxins. The present study reports an outbreak of tetanus in a sheep flock, shortly after ear tagging. Three sheep from a large flock (with a population of 1000 sheep) were presented with signs of: convulsion, limb stiffness, incoordination and trismus (“lock jaw”). There were wounds and scabs in most livestock where ear tags had been attached 1 week prior. Clinical examination revealed tachycardia, dyspnoea with dilated nostrils, mild fever, erected ear pinnae, teeth grinding, mild bloat, muscles rigidity, prolapse of third eyelid and anxiety. According to the history stated by the owner, the case fatality rate of the disease from the beginning was 50% during the outbreak. Necropsy did not reveal any significant finding. Gram‐positive bacilli with terminal spores representing *C. tetani* were isolated in anaerobic cultures which were taken from ear wounds. Procaine penicillin G was administrated at 20 000 IU/kg BW for 5 days, but antiglobulin was not available to treat affected animals. Mortality significantly declined one day after onset of treatment. In this report, the organism was probably introduced by contaminated instruments which were used for ear tagging of sheep. Wound exudation and adhesion following rubbing, created a favourable anaerobic condition for the spores to germinate with production of neurotoxin. Vaccination can protect animals against tetanus, but it does not preclude the need to apply standard hygienic principles when performing management procedures causing wounds. In pasture holding system, many pathogens are present in environment, so tetanus should be considered important in farm animals, because of its high fatality rate and the long course of convalescence.

## Introduction

Tetanus is an acute, often fatal, infectious neuromuscular disease of all farm animals that occurs when a wound becomes infected with spores of *Clostridium tetani* (Wernery *et al*. 2004; Driemeier *et al*. 2007; Muralidharan *et al*. 2010). Almost all mammals are susceptible to tetanus, and the disease has been described in all domestic species, although there is a wide variation in the susceptibility to the tetanus toxin between species. Small ruminants and horses are known to be the most susceptible species, other than humans (Aslani *et al*. 1998; Wernery *et al*. 2004; Driemeier *et al*. 2007). The bacteria are resident in the soil and may be found in animal faeces, because the organism is a common inhabitant of herbivores’ intestinal tracts (Driemeier *et al*. 2007; Muralidharan *et al*. 2010; Constable *et al*. 2017). Tetanus bacteria produce tetanolysin and tetanospasmin. Tetanolysin has the ability to lyse cell membranes, causing tissue damage and stimulating the development of an anaerobic environment (Valgaeren *et al*. 2011; Pugh & Baird 2012). Neurotoxins are then released due to autolysis of the bacterial cell. Tetanospasmin diffuses into the systemic circulation, binds to motor end‐plates and travels up the peripheral nerve trunk via retrograde intra‐axonal transport. This toxin then blocks the spontaneous and nerve impulse‐evoked release of neurotransmitters, resulting in the disinhibition of gamma motor neurons (Singh *et al*. 2000). Consequently it causes muscular rigidity, hyperaesthesia, convulsion and ascending tetany of the skeletal muscles including the respiratory muscles ultimately leading to asphyxia and death (Singh *et al*. 2000; Driemeier *et al*. 2007; Muralidharan *et al*. 2010). Disease is sporadic in general, but outbreaks of tetanus have been described in farm animals occasionally, especially in association with serial herd injections (Driemeier *et al*. 2007; Barbosa *et al*. 2009). Infection occurs as a result of contamination of wounds with spores of *C. tetani*, which sporulates to the vegetative form and produce toxins, if anaerobic conditions are present. Dog bites, fighting, penetration of the oral mucosa by fibrous plant thorns, contaminated umbilical wound, along with persistent skin irritation caused by the constant rubbing by old corroded metal or iron, wood and rope have been identified as common causes for tetanus in ruminants and small ruminants (Aslani *et al*. 1998; Singh *et al*. 2000; Harish *et al*. 2006; Muralidharan *et al*. 2010).

The most frequently described infection sites in farm animals are castration, shearing and injection wounds. The less frequent infection sources such as, surgical contamination, snake bites, dehorning, debudding, tattooing, hoof trimming, docking, surgical interference, vaccination, penetration of object nails and wires, bacterial infection during parturition or manual handing of genitalia, retention of placenta and uterine prolapse, umbilical infections particularly in neonates and ear tagging have also been described in animals (Linnenbrink & Macmichael 2006; Smith & Sherman 2009; Kumar Das *et al*. 2011; Pugh & Baird 2012). This case report describes an outbreak of tetanus in a big sheep flock, shortly after ear tagging.

## Case presentation

**Figure 1:**
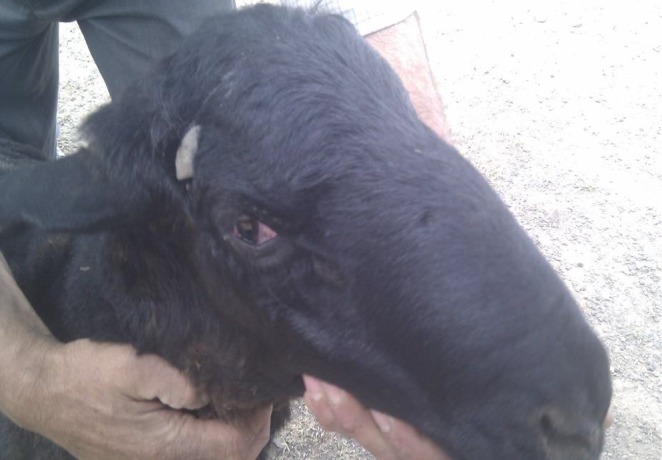
Prolapse of nictitans membrane.

**Figure 2:**
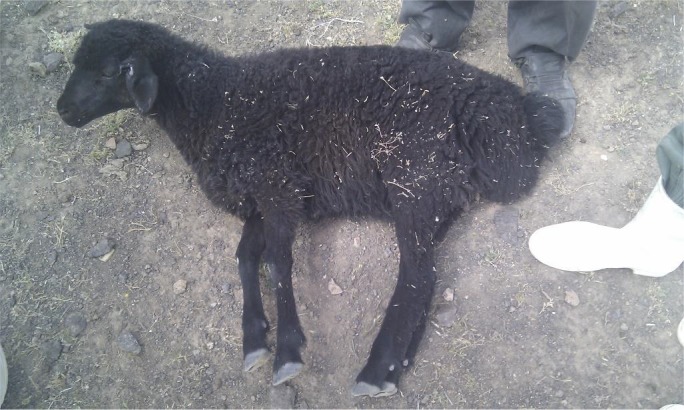
Rigidity of muscles and recumbency.

In May 2012, three sheep from a big sheep flock with 1000 sheep was presented to Veterinary Research and Teaching Hospital (VRTH) of Faculty of Veterinary Medicine, University of Tehran, with signs of: convulsion, stiffness of limbs, incoordination and trismus (“lock jaw”). Rectal temperature in cases was varied and in severe cases rose up to 41°C. Tachycardia and increased respiratory rate together with dyspnoea were other clinical signs noted. Forcing animals to walk or turning the neck caused prolapse of third eye lids (Fig. 1). On closer examination, it was found that there were wounds with scab formation clinging to animals’ ear tags. Mortality was high and about 70 sheep died in the first week of beginning of disease. A farm visit to the affected herd revealed the whole condition and allowed collection of more information about the situation. At the time of the visit about 200 sheep (20%) were affected showing different signs of the disease. Some sheep were dehydrated; and had an S‐shape position in their necks. Tachycardia, dyspnoea and tachypnoea with dilated nostrils, mild fever, erected ears, teeth grinding, anxiety, mild bloat, rigidity in cervical muscles and subsequent recumbency were evident (Fig. 2). There were wounds, scabs and necrosis in the ears of most sheep where ear tags were attached. Prolapse of the third eyelids was observed and became exaggerated with palpation or tapping of the face below the eyes. The owner stated that the ear tagging had only occurred a week before presentation to the hospital as a requirement for animal insurance.

## Results

**Figure 3:**
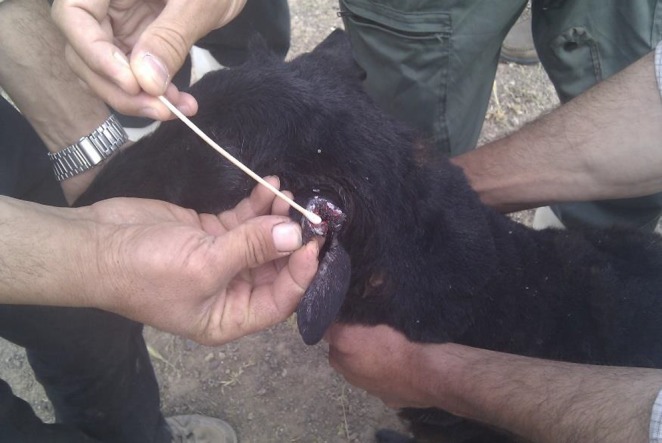
Ear wound due to ear tagging and swabbing.

**Figure 4:**
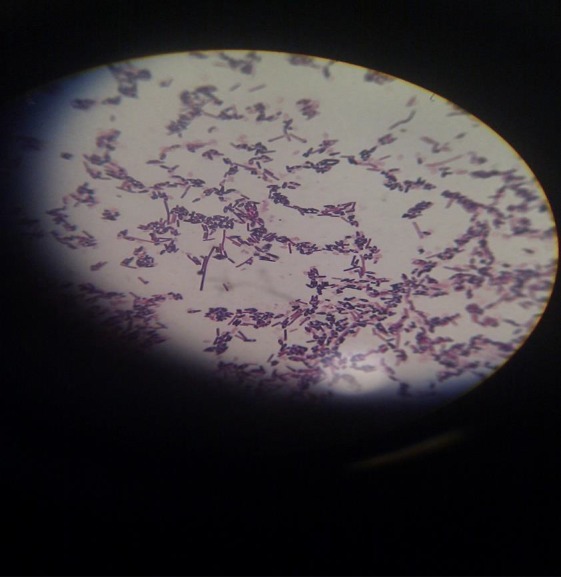
*Clostridium tetani* with terminal spores in certain morphology called ‘tennis racket’.

Bacteriological swabs were taken from depth of the wound sites associated with the ear tags (Fig. 3). Swabs were sent to the laboratory and cultured under aerobic and anaerobic conditions. Swab sampled were cultured anaerobically for 48 h at 37°C in an anaerobic jar. Haemolytic swarming colonies were observed on blood agar media plates. Gram staining of these colonies showed anaerobic bacilli with terminal spores and Gram‐stained slides from the wound depth and necrotic materials on the ears showed the bacilli with spore formation. The outbreak of tetanus was confirmed with the growth of Gram‐positive rods of *C. tetani* with terminal spores with a called ‘tennis racket’ morphology (Fig. 4).

Necropsies were carried out on sheep presented to the VRTH but did not reveal any significant findings. There was no abnormality on microscopic or macroscopic examination of cerebrospinal fluid obtained from the lumbosacral cisterna.

### Differential diagnosis

The diagnosis of tetanus is generally based on the clinical syndrome which it induces. But, depending on the different stages of disease, differential diagnoses must be considered. Laminitis and muscular dystrophy, cause a stilted or stiff gait as seen in early stage of tetanus, but there is no bloat or other signs such as prolapse of third eyelid (Harish *et al*. 2006). In the terminal stages when opisthotonos, recumbency and convulsions are present, polioencephalomalacia must be considered (Smith & Sherman 2009). In this situation, blindness, responding to treatment with thiamine and necropsy finding are helpful. Strychnine poisoning and hypomagnesaemic tetany must also be ruled out by the history and by measurement of serum magnesium (Constable *et al*. 2017). Although, these are less common in sheep, congenital nervous dysfunction, such as myotonia congenita, can produce tetanic spasms, but tetanic convulsion in these diseases are intermittent with episodes of rest between attacks. Rigidity of the neck is also caused by cerebrospinal meningitis and although the animal shows hyperaesthesia, it is generally depressed and hardly excitable (Coetzer & Tustin 2004). So, tetanus can be mistaken with bacterial meningitis because of hyperaesthesia and trismus, but this condition is differentiated by CSF analysis (Smith & Sherman 2009).

In some areas, such as South Africa, tetanus should be differentiated from clinical signs due to some toxic plant that includes: *Sarcostemma viminale* spp. in cattle, sheep and goats; *Cyanthum* spp. in sheep, goats and horses and *Euphorbia mauritanica* in sheep and goats (Coetzer & Tustin 2004). White muscle disease in young ruminants may cause a marked stiffness but no muscle spasm (Coetzer & Tustin 2004; Constable *et al*. 2017). The clinical signs in recumbent sheep suffering from cerebrocortical necrosis may resemble those of tetanus, but the absence of prolapse of the third eyelids and rigidity of leg muscles excludes tetanus (Coetzer & Tustin 2004).

### Treatment

Treatment is difficult, time‐consuming and expensive, and often unsuccessful. It involves the use of tetanus antitoxin to neutralize unbound circulating toxin; penicillin to prevent further growth of *C. tetani;* muscle relaxants to relax rigid muscles; and supportive therapy until the toxin is eliminated or destroyed (Pugh & Baird 2012). The treatment protocol which was used in the present case for remainder of the flock in the flock was intramuscular administration of procaine penicillin G at 20 000 IU/kg BW for 5 days, and because of the inaccessibility and the financial cost to the client, affected animals did not receive antiglobulin.

### Outcome‐follow up

The mortality rate declined significantly just one day after the onset of treatment but continued until a few days later on. However, the condition of all affected animals in this outbreak deteriorated, leading to death and case fatality rate of 50% during the outbreak.

## Discussion

Although tetanus is mainly considered as an individual and sporadic condition, outbreaks have been reported in cattle and sheep (Aslani *et al*. 1998; Driemeier *et al*. 2007). In general, the bacterium has worldwide distribution and is resident in soil and may be excreted in animal faeces (Harish *et al*. 2006; Upadhyay *et al*. 2013). Tetanus should be considered important in farm animals, because of its high fatality rate and the long convalescence (Muralidharan *et al*. 2010). Occurrence of tetanus is variable and depends on some event or set of circumstances such as puncture wounds, obstetrical interventions or performance of routine procedures such as disbudding, dehorning, tattooing, castration, injection, shearing, tagging and hoof trimming (Upadhyay *et al*. 2013).

The highly active neurotoxin is released following multiplication and lysis of the bacteria and may reach the central nervous system by retrograde axonal transport, producing the typical ascending symptoms of tetanus. Through massive toxin production following severe infection, the toxin may directly break the blood–brain barrier, thereby reaching the CNS and produce the descending signs of tetanus (Wernery *et al*. 2004).

In this report, the organism probably introduced by contaminated instruments which were used for repeatedly for ear tagging of sheep, with the wounds following ear tagging being the portal of the entry for the organism and production of toxin. Interestingly, disease signs were more severe in sheep with ear tag closer to the base of the ear. Tetanus is a well‐known disease and can respond to antimicrobial treatment. There are some successful reports of the treatment of tetanus in animals (Harish *et al*. 2006; Kumar Das *et al*. 2011; Bhikane *et al*., 2005 ). In the present case report, despite the lack of antiglobulin against tetanus in treatment regimen, the outcome of the treatment was relatively successful. Bhikane *et al*. (2005) reported that marked improvement was achieved following administration of anti‐tetanus serum in affected animals (Bhikane *et al*. 2005). Although antitoxin is considered the primary choice, Constable *et al*. (2017) stated that tetanus antitoxin is of little value once signs of disease have appeared. Although administration of antitoxin is recommended both parenterally and locally to neutralize the toxin, its effect after appearance of clinical signs is questionable (Upadhyay *et al*. 2013). Two other reports of tetanus due to ear tagging have been recorded in small ruminants (Valgaeren *et al*. 2011).

The first outbreak of tetanus in lamb following ear tag insertion at 7–8 days of age was described by Aslani *et al*. (1998). Valgaeren *et al*. (2011) reported tetanus in a male goat, a week after ear tagging. Insertion of ear tags is routinely done in young ruminants, therefore unhygienic ear tag placement is possible and if there no vaccination of livestock, higher incidence of tetanus outbreak is likely.

This flock was on pasture and whether changeable and windy conditions spread spores of bacteria blowing them onto ear tag wound is not known. Alternatively, inflammation at the ear tag site may causes irritation and makes animal rub their heads and ears on ground or on post and trees. Exudation and adhesion following rubbing, may create a favourable anaerobic condition for the spores to germinate and the produce neurotoxin. Depending on how close the contaminated wound is to the CNS, signs of tetanus become apparent within several days to a week. In this present case, wounds were on ears and within few days clinical disease occurred.

## Source of funding

Not applicable.

## Conflict of Interest

The authors declare that they have no conflicts of interest.

## Ethical statement

The authors confirm that the ethical policies of the journal, as noted on the journal's author guidelines page, have been adhered to and the appropriate ethical review committee approval has been received.

## Contributions

SL conceived the study, clinical diagnosis of disease and helped to draft the manuscript and acted as corresponding author. MH Help in clinical diagnosis and draft the manuscript. MRM Farm visit and clinical diagnosis. MH Microbiological diagnosis of bacteria.

## References

[vms3139-bib-0001] Aslani M.R. , Bazargani T.T. , Ashkar A.A. , Movasaghi A.R. , Raoofi A. & Atiabi N. (1998) Outbreak of tetanus in lambs. The Veterinary Record 142, 518–519. 9618879 10.1136/vr.142.19.518

[vms3139-bib-0002] Barbosa J.D. , Dutra M.D. , Oliveira C.M.C. , Silveira J.A.S. , Albernaz T.T. & Cerqueira V.D. (2009) Outbreak of tetanus in buffaloes (*Buballus bubalis*) in pará, Brazil. Pesquisa Veterinária Brasileira 29, 263–265.

[vms3139-bib-0003] Bhikane A.U. , Yadav G.U. , Karpe A.G. & Ambore B.N. (2005) Tetanus in a Deoni calf ‐ a case report. Intas Polivet 6, 42–43.

[vms3139-bib-0004] Coetzer J.A.W. , Tustin R.C. , (2004). Infectious Disease of Livestock. 2nd edn, Oxford University Press:Cape Town, South Africa.

[vms3139-bib-0005] Constable P.D. , Hinchcliff K.W. , Done S. . (2017). Veterinary Medicine, a Textbook of the Diseases of Cattle, Horses, Sheep, Pigs and Goats. 11th edn P. 1360 Elsevier:St. Louis, MO.

[vms3139-bib-0006] Driemeier D. , Schild A.L. , Fernandes J.C.T. , Colodel E.M. , Correa A.M. , Cruz C.E.F. & Barros C.S.L. (2007) Outbreaks of tetanus in beef cattle and sheep in Brazil associated with disophenol injection. Journal of Veterinary Medicine. A, Physiology, Pathology, Clinical Medicine 54, 333–335. 10.1111/j.1439-0442.2007.00922.x 17650154

[vms3139-bib-0007] Harish B.R. , Chandranaik B.M. , Bhanuprakash R.A. , Jayakumar S.R. , Renukaprasad C. & Krishnappa G. (2006) *Clostridium tetani* infection in goats. Intas Polivet 7, 72–74.

[vms3139-bib-0008] Kumar Das A. , Kumar B. & Kumar N. (2011) Tetanus in a buffalo calf and its therapeutic management. Intas Polivet 12, 383–384.

[vms3139-bib-0009] Linnenbrink T. & Macmichael M. (2006) Tetanus: pathophysiology, clinical signs, diagnosis, and update on new treatment modalities. Journal of Veterinary Emergency and Critical Care 16, 199–207.

[vms3139-bib-0010] Muralidharan J. , Ramesh V. & Saravanan S. (2010) Tetanus in sheep of an organized livestock farm‐ a case report. Indian Journal of Field Veterinarians 5, 43–44.

[vms3139-bib-0011] Pugh D.G. , Baird A.N. , (2012). Sheep and Goat Medicine, 2nd edn Saunders, an imprint of Elsevier Inc:Philadelphia, PA.

[vms3139-bib-0012] Singh A.P. , Tanwar R.K. , Meena D.S. & Gahlot A.K. (2000) Tetanus in a cow‐ a case report. Veterinary Practitioner 1, 137–138.

[vms3139-bib-0013] Smith M.C. & Sherman D.M. (2009) Goat Medicine. 2nd edn Wiley‐Blackwell: Hong Kong, Printed in Singapore.

[vms3139-bib-0014] Upadhyay S.R. , Hussain K. & Singh R. (2013) Bovine neonatal tetanus: a case report. Buffalo Bulletin 32, 18–20.

[vms3139-bib-0015] Valgaeren B. , De Schutter P. , Pardon B. , Eeckhaunt V. , Boyen F. , Van Immerseel F. & Deprez P. (2011) Thermic dehorning and ear tagging as atypical portals of entry of *Clostridium tetani* in ruminants. Vlaams Diergeneeskundig Tijdschrift 80, 351–354.

[vms3139-bib-0016] Wernery U. , Ui‐Haq A. , Joseph M. & Kinne J. (2004) Tetanus in a camel (*Camelus dromedaries*) – a case report. Tropical Animal Health and Production 36, 217–224. 15080538 10.1023/b:trop.0000016835.02928.28

